# The effect of statins exposure during pregnancy on congenital anomalies and spontaneous abortions: A systematic review and meta-analysis

**DOI:** 10.3389/fphar.2022.1003060

**Published:** 2022-09-29

**Authors:** Ayala Hirsch, Natali Ternovsky, Donna R. Zwas, Reut Rotem, Offer Amir, Bruria Hirsh Raccah

**Affiliations:** ^1^ Department of Obstetrics and Gynecology, Shaare Zedek Medical Center, Affiliated with the Hebrew University School of Medicine, Jerusalem, Israel; ^2^ Division of Clinical Pharmacy, Institute for Drug Research, School of Pharmacy, Faculty of Medicine, Hebrew University of Jerusalem, Jerusalem, Israel; ^3^ Linda Joy Pollin Cardiovascular Wellness Center for Women, Department of Cardiology, Hadassah University Hospital, Jerusalem, Israel; ^4^ Department of Cardiology, Hadassah University Hospital, Jerusalem, Israel

**Keywords:** statins, hydroxymethylglutaryl-coenzyme a (HMG-CoA) reductase inhibitors, congenital anomalies, cardiac anomalies, spontaneous abortion

## Abstract

**Objective:** To assess the effect of statin exposure during pregnancy on congenital anomalies and spontaneous abortions.

**Data sources:** Electronic databases were searched from inception to January 2022.

**Study Eligibility Criteria:** Cohort studies and randomized controlled trials (RCTs) evaluate the effect of treatment with statins on congenital anomalies in general and cardiac malformations in particular. Studies evaluating spontaneous abortions were included as a secondary outcome.

**Study appraisal and synthesis methods:** Pooled odds ratio was calculated using a random-effects model and meta-regression was utilized when applicable.

**Results:** Twelve cohort studies and RCTs were included in the analysis. Pregnancy outcomes of 2,447 women that received statins during pregnancy were compared to 897,280 pregnant women who did not. Treatment with statins was not associated with a higher risk of overall congenital anomalies (Odd Ratio = 1.1, CI (0.9–1.3), *p* = 0.33, I2 = 0%). Yet, cardiac malformations were more prevalent among neonates born to statins users (OR = 1.4, CI (1.1–1.8), *p* = 0.02, I^2^ = 0%). The risk was higher when exposure occurred during the first trimester. This finding was statistically significant in cohort studies, but not in RCTs. Statin treatment was also associated with a higher rate of spontaneous abortions (OR = 1.5, CI (1.1–2.0), *p* = 0.005, I^2^ = 0%). In meta-regression analysis, no significant association between lipophilic statins and the rate of congenital anomalies was found.

**Conclusion:** Overall, treatment with statins during pregnancy was not associated with an increased risk of congenital anomalies. A slight risk elevation for cardiac malformation and spontaneous abortions was seen in cohort studies but not in RCTs.

**Systematic Review Registration:** clinicaltrials.gov, identifier [CRD42020165804 17/2/2020]

The meta-analysis was presented online at 42nd annual meeting of SMFM. January 31-5 February 2022.

## 1 Introduction

Hydroxymethylglutaryl-coenzyme A (HMG-CoA) reductase inhibitors, commonly called statins, are widely used for hypercholesterolemia and have been shown to reduce mortality and morbidity of cardiovascular disease. (Ray and Cannon, 2005; Reiner, 2013; Catapano et al., 2016).

As the incidence of obesity and cardiovascular disease surges in younger populations, and as more women commonly delay pregnancy later in life, the use of statins in patients of reproductive age has increased. (Hayes et al., 2020). Statins are also indicated in young women with familial hypercholesterolemia and in patients with polycystic ovary syndrome. (Cassidy-Vu et al., 2016), (Versmissen et al., 2009) Of note, there is promising data on treating uteroplacental insufficiency disorders, including preeclampsia and fetal growth restriction, with pravastatin. (Akbar et al., 2021a), (Vahedian-Azimi et al., 2021a) The expanding indications for statin treatment (Stone et al., 20132014) contribute to the rising number of women who present with an established pregnancy or with a wish to become pregnant while treated with statins. (Cooper et al., 2004)., (Kulaga et al., 2009)

Discontinuation of lipid-lowering therapy during pregnancy may have adverse consequences for both fetus and mother. (Napoli et al., 1999; Avis et al., 2009; Van Der Graaf et al., 2010; Thobani et al., 2021). Hypercholesterolemia during gestation has been shown to be associated with preterm labor (Mudd et al., 2012) preeclampsia and fetal growth restriction (Roes et al., 2005; Ziaei et al., 2012; Alahakoon et al., 2020). At the same time, cholesterol is essential during embryonic development: during the first weeks of gestation, fetuses depend on maternal cholesterol (Baardman et al., 2013) and maternal HMG-CoA reductase activity is important for placental development (Kenis et al., 2005), (Lecarpentier et al., 2012).

Statins have been designated as pregnancy category X by the U.S. Federal Drug Administration (FDA) (Register, 2014) based on animal models, case reports, patient registries, and small cohort studies. (Edison and Muenke, 2004a; Edison and Muenke, 2004b; Edison and Muenke, 2004c; Gibb et al., 2005; Pollack et al., 2005; Ofori et al., 2007; Petersen et al., 2008; Taguchi et al., 2008; Bateman et al., 2015). Yet, recent studies did not find a link between congenital anomalies and statin exposure in pregnancy, and on 20 July 2021, the FDA requested the removal of its warning against using statins in pregnant patients. (Statins, 2022).

Because of the growing number of women eligible for statin therapy and the importance of continuity of statin therapy for both mothers and fetuses, and the promising evidence of the role of statins in treating and preventing obstetrical complications, the implications of gestational exposure to statins is an issue of significant clinical importance. The purpose of this meta-analysis is to determine whether the use of statins increases the incidence of congenital malformations or spontaneous abortions.

## 2 Materials and methods

### 2.1 Information sources

This systematic review followed the Preferred Reporting Items for Systematic Reviews and Meta-Analyses (2020) framework guidelines (PRISMA) (Page et al., 2021) and the Meta-analysis Of Observational Studies in Epidemiology (MOOSE) guidelines (MOOSE, 2019). (Supplementary Tables S1,S2)

We conducted three systematic database searches: the first included articles from 1953 to January 2021, the second included articles published from the last search to April 2021 and the third included article from the last search to January 2022. The search included PubMed (MEDLINE), EMBASE, Clinical Trials Registry (clinicaltrials.gov) and The Cochrane Library. Language restrictions were not set.

### 2.2 Search strategy

The search strategies incorporated index terms (Mesh) and free text words for the search concepts: pravastatin, atorvastatin, rosuvastatin, pregnancy combined by “AND”; and in each domain, the terms were combined by “OR” (Supplementary Table S3) The first domain contained terms on statins (including synonyms and abbreviations such as HMG-CoA reductase inhibitors), the second domain related to pregnancy.

The detailed protocol is documented online in the International Prospective Register of Systematic Reviews registry (CRD42020165804). Because this study was a review and meta-analysis, no internal review board approval was required. All randomized controlled trials included in the analysis declared that they appropriately registered in a clinical trials registry.

### 2.3 Eligibility criteria

In the search strategy, we included randomized controlled trials (RCTs), non-randomized controlled clinical trials, prospective and retrospective comparative cohort studies, and case-control studies. Every study that included women treated with statins during pregnancy was analysed. Duplicated reports, case reports, case series, cross-sectional studies, pharmacokinetic studies in healthy adults, animal studies, reviews, expert opinion, editorials, letters to the editor, comments, and studies with a high risk of bias were excluded.

### 2.4 Study selection and data extraction

Two investigators independently identified and extracted articles for potential inclusion using the Rayyan QCRI web application for systematic review. (Ouzzani et al., 2016). Disagreements were resolved by referral to a third reviewer. The full texts of the resulting references were then retrieved and analyzed. If more than a single study published data from the same cohort, we included the report with the higher quality according to Newcastle-Ottawa Quality Assessment Scale (GA Wells et al., 1932) to avoid overlap.

Exposure to statins during pregnancy was defined as exposure to any dose and in any trimester of pregnancy. The primary outcomes included overall congenital anomalies: major and/or minor, and in particular cardiac malformations. The secondary outcome included spontaneous abortion which was defined as abortions, miscarriages or stillbirth.

Twin pregnancy results were excluded

Data from included studies were extracted by a single reviewer and subsequently evaluated by the second reviewer. For studies that did not report the outcomes, we contacted the authors and requested the missing data.

### 2.5 Quality assessment and risk of bias

Risk of bias and quality were assessed using the Newcastle-Ottawa scale (GA Wells et al., 1932) (NOS) for assessing quality of observational studies. The scale is based on eight criteria and provides a star rating score ranging from 0 (high risk for bias) to 9 (low risk for bias). A 5-star rating and below was designated high risk of bias, 6-7 stars intermediate risk of bias, and 8-9 stars low risk of bias. Randomized controlled studies were evaluated by the Cochrane Collaboration’s Risk of Bias Tool. (Higgins et al., 2011). Summary assessments of risk of bias were derived for each study. Assessments were carried out independently by 2 investigators.

Publication bias was estimated visually by funnel plot and with Egger’s regression test to measure funnel plot asymmetry. In the funnel plot, studies with a large number of participants appear toward the top of the graph and generally cluster around the mean effect size and a smaller SE. Studies with a small number of participants appear toward the bottom of the graph and tend to be spread across abroad range of effects size values and SEs.

### 2.6 Data synthesis and statistical analysis

Meta-analysis and Meta-regression were performed using Comprehensive Meta-Analysis software. Random-effect pooled odds ratio (OR), based on the inverse-variance approach, was calculated with the corresponding 95% confidence intervals (CIs). Heterogeneity was assessed using the I^2^ statistic. I^2^ values of 25%, 50%, and 75% represented low, medium, and high heterogeneity respectively. (Higgins and Thompson, 2002) Statistical significance was defined using a 2-sided a of <0 .05, and interpretations of clinical significance emphasized CIs. Meta-regression uses trial-level covariates to detect possible sources of heterogeneity and to relate the size of the reported effect (e.g., congenital malformations) to one or more characteristic of the studies in the analysis. (Thompson and Higgins, 2002) Meta-regression is weighted to assess within-trial exposure effects and between-trial variances using Comprehensive Meta-Analysis software. Subgroups’ analyses were made in order to identify covariates that may affect the results: Gestational age at exposure, first trimester vs. second and third trimesters, and lipophilic vs. non-lipophilic statins. Lipophilic statins included atorvastatin, simvastatin, lovastatin, fluvastatin, cerivastatin, and pitavastatin. Non-lipophilic statins included rosuvastatin and pravastatin. A subgroup analysis evaluating RCT and Cohort studies separately was performed in order to assess whether study design had an impact on effect size and to estimate the robustness of our primary findings.

## 3 Results

### 3.1 Study selection

**FIGURE 1:**
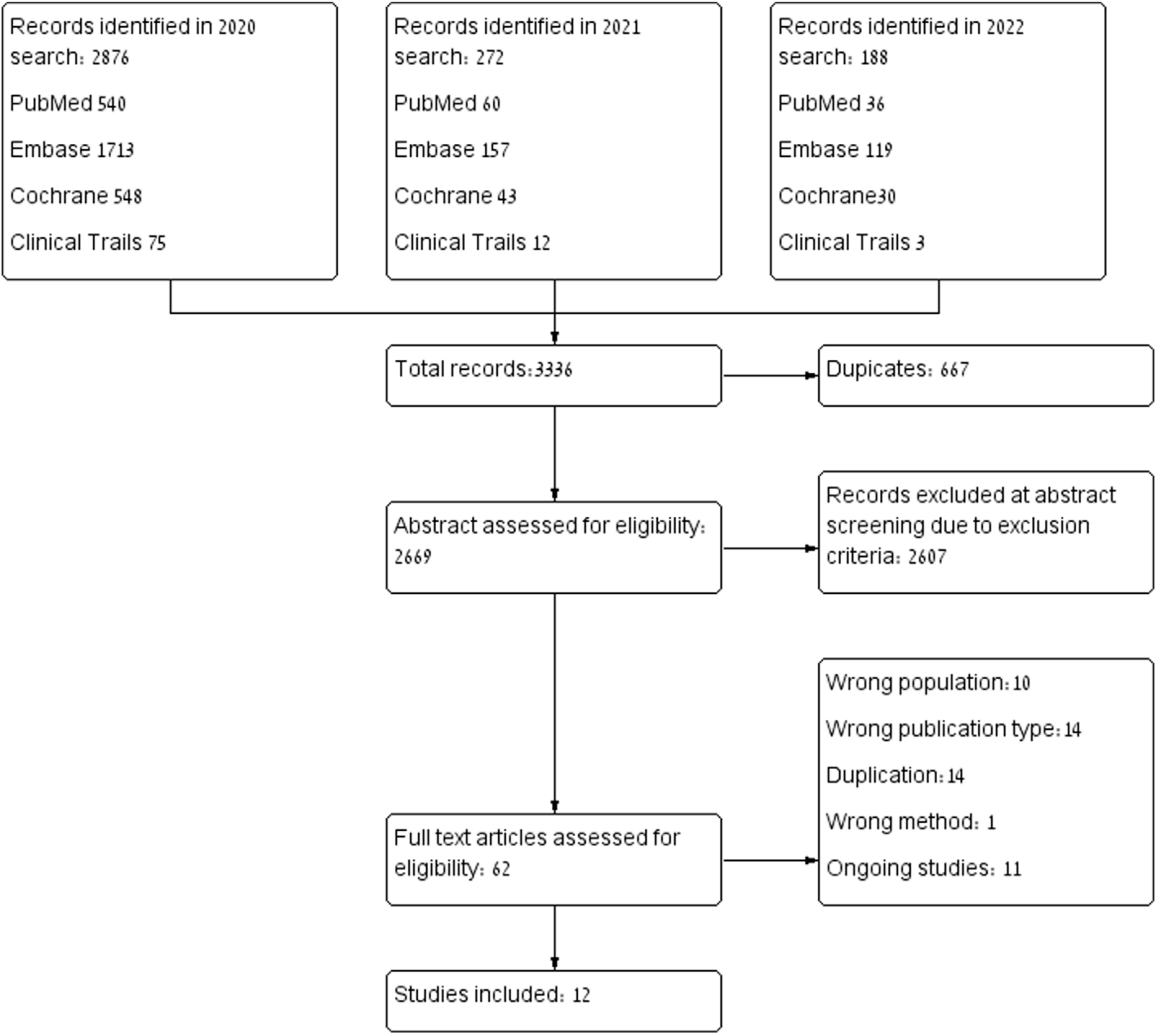
Publication selection process.

The database search yielded 3,336 citations: PubMed (n = 636), Embase (*n* = 1989), Cochrane Library (*n* = 621), clinicaltrials.gov (*n* = 90). In total, 667 were identical duplicates, and excluded. After abstract assessment, sixty-two articles were extracted for full-text review. Fifteen articles were excluded because of publication type, study design, wrong methods, wrong outcome or wrong population. (Pollack et al., 2005), (Kadioglu et al., 2020; Kupferminc et al., 2021; Toleikyte et al., 2011; Christensen et al., 2021; Costantine, 2022; Akbar et al., 2021b; Lefkou et al., 2020; Jurisic et al., 2021; Desai et al., 2017; Colvin1 et al., 2010; Ruys Titia et al., 2014)Studies involving animals or human placenta were excluded, and only clinical studies were included. Due to a lack of standardization in studies investigating biomolecular markers, those studies or results were excluded as well. The selection process is illustrated in Figure 1. Ultimately, twelve studies were included in the analysis, with a total of 897,280 pregnant women. Of them, 2,447 were treated with statins and 894,833 were not treated with statins.

### 3.2 Study characteristics

The studies were published between 2007 and 2021 and originated from the United States (*n* = 4), Canada (*n* = 2), United Kingdom (*n* = 2), Indonesia (*n* = 1), Greece (*n* = 1), Taiwan (*n* = 1) and multicentre over Europe (*n* = 1). Four studies were RCT’s (Costantine et al., 2016; Ahmed et al., 2020; Deviana et al., 2020), (Costantine et al., 2021) and eight studies were cohort studies (Lefkou et al., 2016; McGrogan et al., 2017; Lee et al., 2018; Chang et al., 2021; Winterfeld et al., 2013), (Taguchi et al., 2008; Bateman et al., 2015), (Ofori et al., 2007).

Five studies were performed on women who had preeclampsia or were at high risk for obstetrical complications involving uteroplacental insufficiency (Costantine et al., 2021), (Costantine et al., 2016; Ahmed et al., 2020; Deviana et al., 2020), (Lefkou et al., 2016), two of these were performed on women who had also antiphospholipid syndrome and fetal growth restriction, one study was performed on women who had early onset intra-uterine fetal growth restriction, and one on women with abnormal Dopplers in the uterine artery.

Concerning the type of statin used, pravastatin was used in all twelve studies, simvastatin and atorvastatin were used in seven studies, rosuvastatin and fluvastatin in five studies, lovastatin in four studies and cerivastatin in three studies (Table 1).

**TABLE 1:** key characteristics of the included trials.

Author and year	Study location	Study period	Study design	Population characteristics	Intervention vs. control group	Number of women statins/controls	Time of exposure
Ofori, 2007	Canada	1997-2003	Cohort	Med-Echo and ISQ databases	Atorvastatin, Fluvastatin, Lovastatin, Pravastatin, Simvastatin/Exposure to statins between 1 year before and 1 month before pregnancy	153/106	First trimester
Taguchi, 2008	Canada	1998-2005	Cohort	Teratogen information service	Atorvastatin, Simvastatin, Pravastatin, Rosuvastatin/Agents known to be non-teratogenic	64/64	First trimester
Winterfeld, 2013	Multinational centers in Europe	1990-2009	Cohort	Teratology Information Services	Simvastatin, Atorvastatin, Pravastatin, Rosuvastatin, Fluvastatin, Cerivastatin/Agents known to be non-teratogenic	249/249	First trimester
Bateman, 2015	United States	2000-2007	Cohort	Medicaid Analytic eXtract	Simvastatin, Lovastatin, Pravastatin, Fluvastatin, Atorvastatin, Cerivastatin, Rosuvastatin/No treatment	1,152/885,844	First trimester
Lefkou, 2016	Greece	2013-2015	Cohort	APLS with PET and/or IUGR	Pravastatin 20 mg + Aspirin + LMWH/Aspirin + LMWH	10/11	Second-third trimester
Costantine, 2016	United States	2012-2014	RCT	High risk for PET	Pravastatin 10 mg/placebo	10/10	Second trimester
McGrogan, 2017	United Kingdom	1992-2009	Cohort	General Practice Research Database	Simvastatin, Atorvastatin, Cerivastatin, Rosuvastatin, Pravastatin, Fluvastatin and combination/No exposure to statins	281/2,643	First trimester
Ming-Sum Lee, 2018	United States	2003-2014	Cohort	Pharmacy dispensing records	Atorvastatin, Lovastatin, Pravastatin, Simvastatin/No exposure to statins	279/1,160	First trimester
Ahmed, 2019	United Kingdom	2011-2014	RCT	Early-onset of PET	Pravastatin 40 mg/placebo	30/32	Second-third trimester
Soraya Riu, 2019	Indonesia	2019	RCT	High risk for PET	Pravastatin 20*2 + Aspirin 80/Aspirin 80	18/15	Second trimester
Chung Chang, 2021	Taiwan	2004-2014	cohort	Taiwan National Health Insurance Research Database	Atorvastatin, Rosuvastatin, Lovastatin, Simvastatin, Fluvastatin, Pravastatin, combination/No exposure to statins or other teratogenic drugs	469/4,690	No information
Costantine, 2021	United States	No information	RCT	high risk for PET	Pravastatin 20*1/placebo	10/10	Second trimester

RCT, randomized controlled trial; LMWH, low molecular weight heparin; PET, preeclampsia; IUGR, intrauterine growth restriction; APLS, antiphospholipid syndrome.

In six studies (Winterfeld et al., 2013)^,^ (McGrogan et al., 2017; Lee et al., 2018), (Taguchi et al., 2008; Bateman et al., 2015), (Ofori et al., 2007), women were exposed to statins during the first trimester of pregnancy and in five studies (Costantine et al., 2016; Ahmed et al., 2020; Deviana et al., 2020), (Costantine et al., 2021) statins were used during second to third trimester of pregnancy. One study did not include specific information about the time of exposure. One study (Chang et al., 2021) did not include information about the time at exposure.

Table 1 shows a summary of the key characteristics of the included trials.

Details regarding the specific malformations were reported for six of the twelve studies. Winterfeld et al. (2013) divided malformation types into major and minor birth defects, but as other studies did not separate malformations based on major or minor classifications, for this meta-analysis malformations were combined. Supplementary Table S4 summarizes a list of the congenital malformations that were reported in these studies. Supplementary Table S5 summarizes the numbers of events or odds ratio that were used in each study.

### 3.3 Risk of bias in included studies

The overall risk of bias among nonrandomized studies according to Newcastle-Ottawa Quality Assessment Scale (NOS) was 8.1. The overall risk of bias of three randomized controlled studies evaluated by the Cochrane Collaboration’s Risk of Bias Tool was low. Risk of bias assessment is summarized in Supplementary Table S6 and Supplementary Figure S1.

Publication bias was calculated for the congenital malformation analysis, (including 11 studies) and for the analysis of cardiac anomalies (including 10 studies). Visual inspection of the funnel plot (Supplementary Figures S2,S3) and Egger’s regression test did not reach significance for publication bias (*p*-value = 0.4 and *P*- value = 0.8 respectively).

### 3.4 Synthesis of results

#### 3.4.1 Congenital anomalies

**FIGURE 2:**
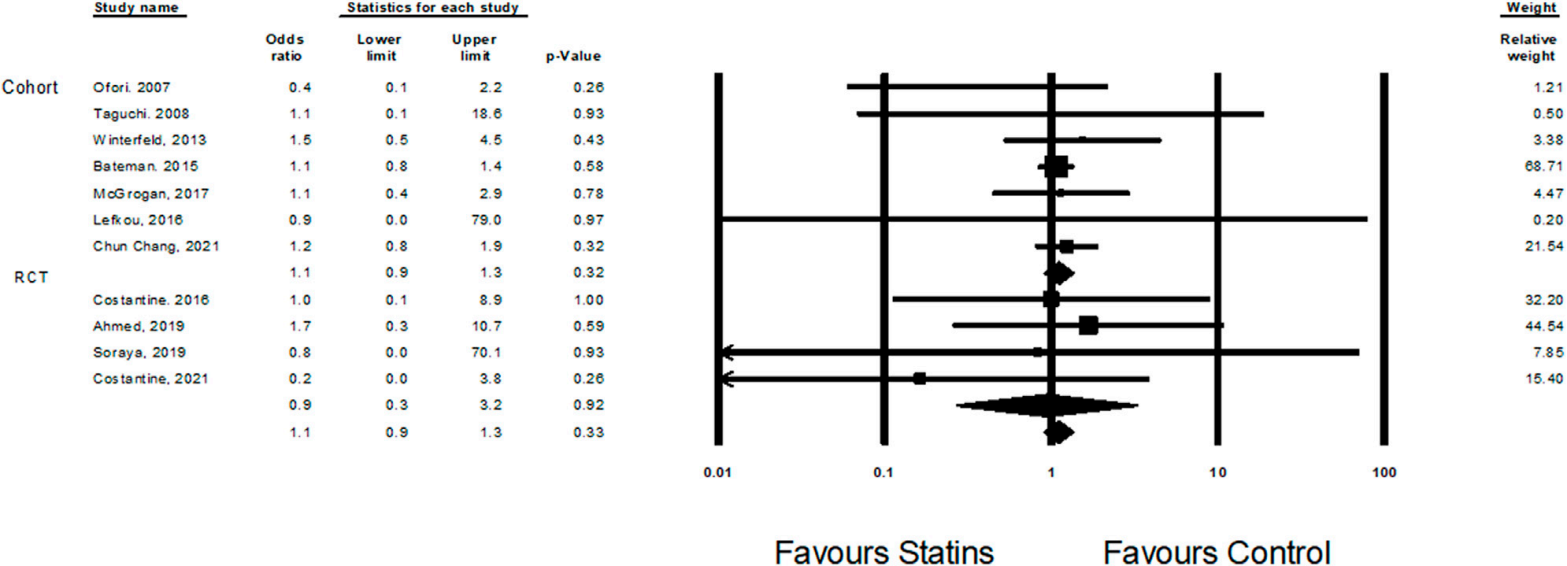
The Odd ratios for congenital anomalies following statins treatment versus control treatment.

Eleven studies (Taguchi et al., 2008; Bateman et al., 2015), (Ofori et al., 2007), (Costantine et al., 2016; Lefkou et al., 2016; McGrogan et al., 2017; Ahmed et al., 2020; Deviana et al., 2020; Chang et al., 2021; Winterfeld et al., 2013; Costantine et al., 2021) examined the effect of statin treatment on congenital anomalies. Overall, treatment with statins was not associated with increased risk of congenital anomalies (Odd Ratio = 1.1, CI (0.9–1.3), *p* = 0.33, I2 = 0%) (Figure 2).

#### 3.4.2 Cardiac malformation

**FIGURE 3:**
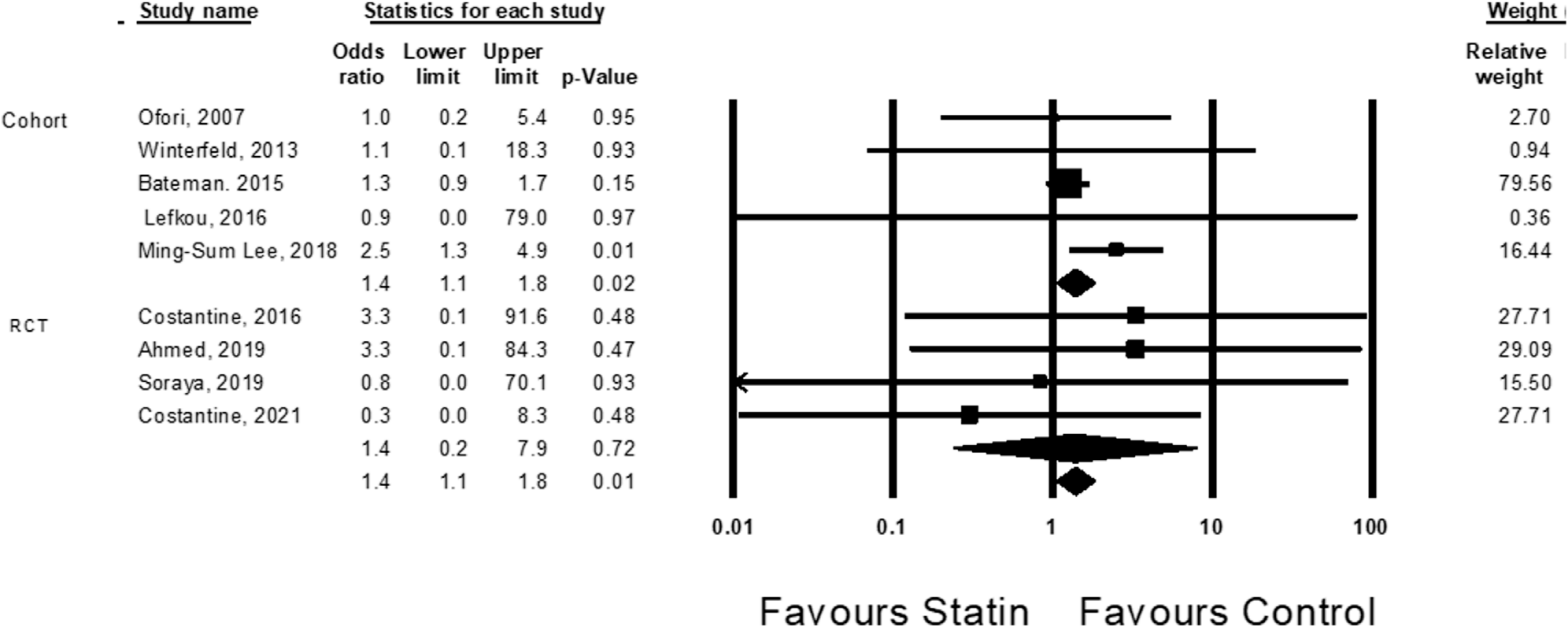
The Odd ratios for cardiac anomalies following statins treatment versus control treatment.

Cardiac malformations were evaluated in nine studies (Ofori et al., 2007; Bateman et al., 2015; Costantine et al., 2016; Lefkou et al., 2016; Lee et al., 2018; Ahmed et al., 2020; Deviana et al., 2020; Costantine et al., 2021; Winterfeld et al., 2013). Treatment with statins was associated with an elevated risk of cardiac malformation (OR = 1.4, CI (1.1–1.8), *p* = 0.01, I^2^ = 0%). The association was statistically significant in cohort studies, but not in RCT studies (Figure 3).

**FIGURE 4:**
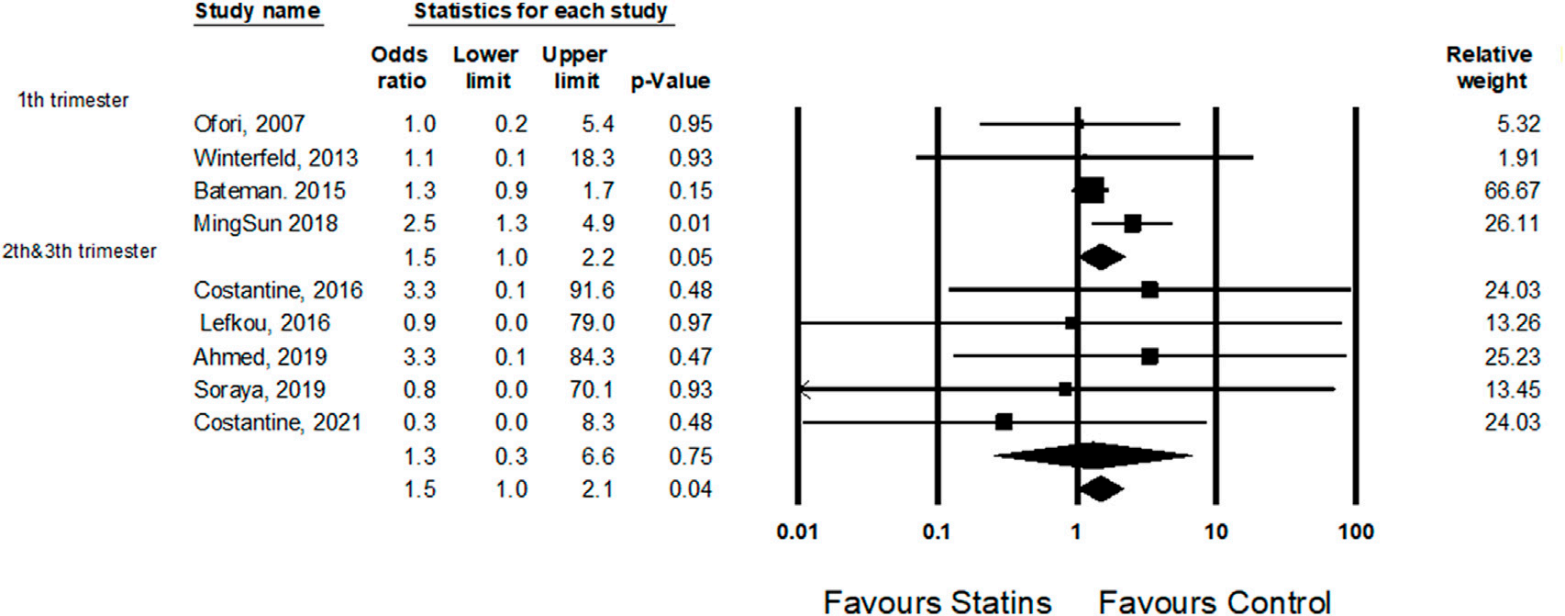
A subgroup analysis: the odds ratio for cardiac anomalies following statins treatment in the first trimester comparing statins treatment in the second and third trimesters.

A subgroup analysis revealed a difference in the odds ratio for cardiac malformations for those exposed to statins in the first trimester compared to those exposed in the second and third trimester (1.5, 95% CI 1.0–2.2, *p*-Value 0.05 and 1.3, 95% CI 0.3–6.4, *p*-Value 0.76, respectively) (Figure 4).

The small increase in the risk for cardiac malformations is primarily driven by the study of Ming-Sum Lee et al. (2018); when this study is excluded, this finding is not statistically significant (OR = 1.2, CI (0.9–1.7), *p* = 0.14, I^2^ = 0%) (Supplementary Figure S4).

#### 3.4.3 Spontaneous abortions

**FIGURE 5:**
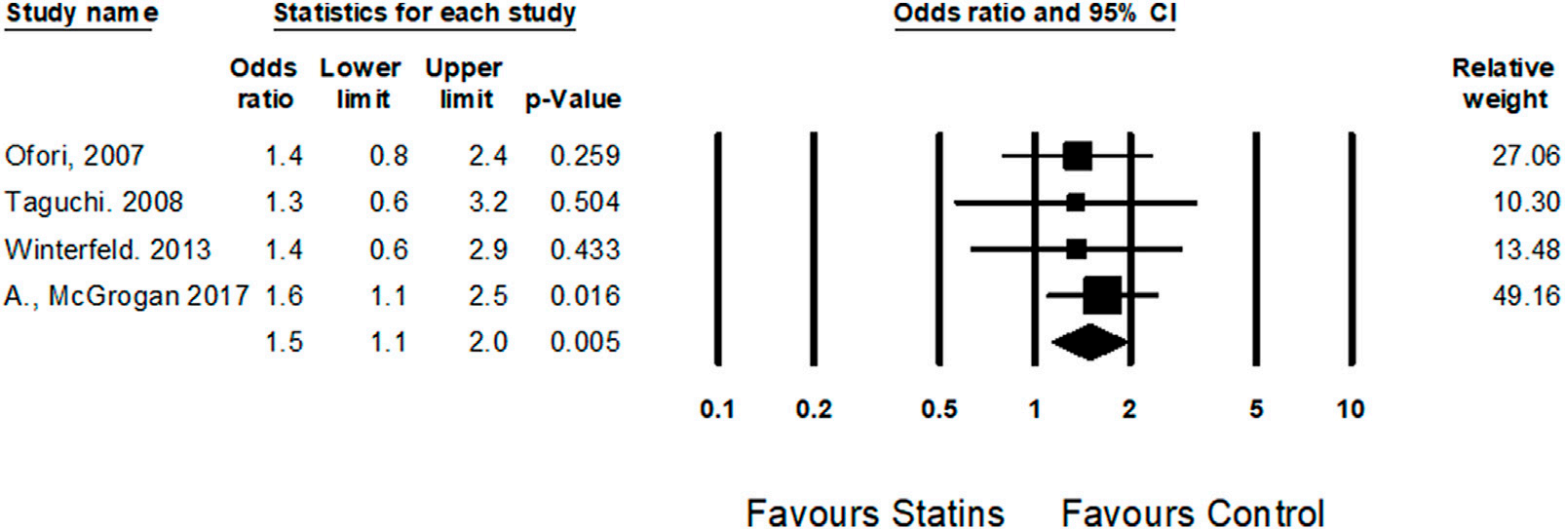
The Odd ratios for spontaneous abortions following statins treatment versus control treatment subgroup by trimester expose.

Four cohort studies (Ofori et al., 2007; Winterfeld et al., 2013; Taguchi et al., 2008; McGrogan et al., 2017) examined the risk of spontaneous abortions among statins users during the first trimester. Statin treatment was associated with an increased risk of spontaneous abortions (OR = 1.5, CI (1.1–2.0), *p* = 0.005, I^2^ = 0%) (Figure 5).

#### 3.4.4 Meta-regression

Three meta-regression analyses were conducted to evaluate the association between treatment with lipophilic statins, the association with the incidence of gestational diabetes and pregnancy-induced hypertension and outcomes. No statistically significant association was found between congenital anomalies or cardiac malformations and the use of lipophilic vs. non-lipophilic statins, maternal diabetes and hypertension between treatment and control groups (*R* ^2^ = 0). (Supplementary Figures S5–S10).

## 4 Comments

### 4.1 Principal findings

In this systematic review and meta-analysis, we found that exposure to statins during pregnancy was not associated with an overall increased risk for congenital malformations. However, treatment with statins during pregnancy was associated with an elevated risk of cardiac malformations. Sub-group analysis demonstrated that this increased risk was only in cohort studies and not in RCTs. Moreover, the small increase in the risk for cardiac malformations was mainly due to one specific cohort study of Ming-Sum Lee et al. (2018). A second analysis that excluded this study did not find a statistically significant increased risk for cardiac malformation. Another subgroup analysis revealed that the increased risk for cardiac malformations was statistically significant only after exposure in the first trimester and not if the exposure occurred later in pregnancy. Moreover, in several of the studies that reported an increased risk of cardiac malformation with statins therapy, there was concurrent exposure to additional drugs such as antagonists to angiotensin II, ACEI (Angiotensin Converting Enzyme inhibitor), ARB (Angiotensin II Receptor Blocker), and anti-epileptic drugs, which are now known to have a teratogenic or toxic effect. For example, the study of Winterfeld et al. (2013) which reported fetal cardiomegaly with rosuvastatin exposure, also included exposure to valsartan. McGrogan et al. (2017) reported that over 30% of the additional medications prescribed during pregnancy in the setting of statin exposure included ACEI and ARB. Supplementary Table S7 summarizes the percentage of cases where ACEI, ARB and anti-epileptic drugs were used in studies that were included in the analysis of cardiac malformations.

Conversely, preclinical studies exposing mouse embryonic stem cells to statins showed changes in genes expression such as NKX 2.5 andα/β-myosin heavy chain, which potentially can affect embryonic cardiac development. (Jyoti and Tandon, 2015). The studies in our meta-analysis reported various cardiac malformations, including coarctation of the aorta, cardiomegaly, atrial septal defect, ventricular septal defect, and cardiac arrhythmia. (Supplementary Table S4 in yellow).

Statin treatment was also found to be associated with a higher rate of spontaneous abortions in the first trimester in cohort studies. Thus, the increased risk might result from background characteristics of the population including age, metabolic condition, and co-morbidities that may predispose pregnancies toward miscarriage rather than statin therapy.

### 4.2 Comparison with existing literature

The safety profile of statins exposure during pregnancy is not well defined. The United States Food and Drug Administration labeling recommends against the use of statins during pregnancy based on the essential role of cholesterol during pregnancy and animal data showing teratogenic potential at high doses. (Edison and Muenke, 2004a), (Edison and Muenke, 2004c) Therefore, the current practice suggests the discontinuation of statins when trying to conceive.

Human data assessing the use of statins during pregnancy is scarce and inconsistent and is derived primarily from patient registries, and case reports. (Edison and Muenke, 2004a; Edison and Muenke, 2004b; Edison and Muenke, 2004c; Gibb et al., 2005; Pollack et al., 2005; Ofori et al., 2007; Petersen et al., 2008; Taguchi et al., 2008; Bateman et al., 2015). For example, case reports from 2014 suggested that lipophilic statins may increase the risk of the congenital central nervous system and limb anomalies (Edison and Muenke, 2004c), whereas a case series analysis from the National Birth Defects Prevention Study failed to observe the same distribution of defects. (Petersen et al., 2008). Three reviews and meta-analyses regarding the safety of statins in pregnancy were published, in 2012, 2014, and 2021. The results of the first one (Kusters et al., 2012) indicated that statins were unlikely to be teratogenic. However, this conclusion was based solely on three cohort studies. The second meta-analysis (Zarek and Koren, 2014) included six studies and found no significant difference in overall birth defects (RR = 1.15; 95%CI: 0.75–1.76); cardiac malformations were not analyzed separately. In addition, the second meta-analysis found a significant increase in the rate of spontaneous abortions (RR = 1.35; 95%CI: 1.04–1.75) but the authors suggested that it is more likely to reflect the preexisting conditions of women treated with statins which may predispose them to miscarriage. The third meta-analysis (Vahedian-Azimi et al., 2021b) also did not find a significant increase in birth defects after statin therapy but they found an increased risk for cardiac anomalies. However, this meta-analysis included only six studies that reported congenital anomalies as an outcome and only two that reported cardiac anomalies. This compares to eleven studies and nine studies respectively in our meta-analysis. No meta-regression or sensitivity analyses were performed, nor did they analyze spontaneous abortions.

Despite the recommendation that treatment with statins in pregnancy should be avoided, recent clinical studies have suggested that statin use during pregnancy may reduce the risks of obstetrical complications such as preeclampsia and fetal growth restriction. (Vahedian-Azimi et al., 2021a). Patients with familial hyperlipidemia or coronary artery disease may be at significant risk even with cessation of statin only during pregnancy since pregnancy itself is a significant risk factor for acute myocardial infarction. (James et al., 2006), (Nallapati and Park, 2021) Considering the clear clinical benefit of statin therapy and the growing evidence of statins’ potential benefit in preventing and treating obstetrical complications, there may indeed be a role for statins in pregnancy, in particular in the second and third trimester.

### 4.3 Strengths and limitations

Our meta-analysis has several important strengths. First, this meta-analysis is the largest meta-analysis to date, including all available published data with systematic analysis according to accepted guidelines. We conducted a thorough and extensive search of all available evidence and used structured methods for the collection, evaluation, and reporting of our findings. Second, the meta-analysis included a larger number of RCTs compared to the previous meta-analyses published on this topic, and all studies described a high treatment adherence. This meta-analysis included additional analyses beyond those published on the same topic. We conducted a meta-regression to identify covariates, including lipophilic statin rate, gestational diabetes, and hypertension, that may affect the association between statins exposure during pregnancy and the risk for congenital anomalies. We also conducted subgroup analysis by the type of study (RCT, Cohort) and gestational age at exposure to evaluate whether study design or gestational age had an impact on the effect size.

Our meta-analysis also has several important limitations. Despite including twelve clinical human studies, our review is limited by the sparse clinical data that is available on statin use in pregnancy. Because eight studies included in this meta-analysis are cohort studies, selection bias should be considered. In some of the cohort studies included in this meta-analysis, data on medication exposure was collected by interviews or follow-up on prescription dispensing and database linking. Mothers of infants with a congenital malformation or who underwent spontaneous abortions may be more likely to recall and to associate between exposure to medications during pregnancy and offspring morbidity. Furthermore, prescription dispensing does not necessarily indicate intrauterine exposure to statins. Additionally, because of the large variability in some of the studies, and small numbers of studies in the analyses, the estimated random effect variance is very small, meaning that large studies with small variance are favored and have large relative weights. To fully reflect the impact and understand if the pooled findings are not a copy of a single study, we made a sensitivity analysis without the largest study (Bateman et al., 2015), The pooled OR was similar to the original OR with the Batemen study and fell inside the original 95th confidence interval (Supplementary Figure S11). We also performed a quality assessment for bias risk using the NOS, which resulted in a low probability of selection bias. Another limitation is that study-level meta-analysis does not include the adjustment for all covariates that may affect the risk for congenital malformations and spontaneous abortions; however, we used adjusted effect sizes meta-analysis. The cohorts included in this meta-analysis were geographically and racially diverse, which can potentially broaden the generalizability of our results yet magnify the difference in management and background characteristics. In this analysis, we pooled together studies with adjustments for confounders including background diseases, obstetric characteristics, and conditions, drugs dispensed to the mother, socioeconomic information, education, etc. Therefore, there is the possibility that the results may not reflect the effect size and may be exposed to different sources of bias. The heterogeneous studies represent real-life conditions and pooling the results together may give a clue on the direction of the outcomes.

## 5 Conclusion and implications

Overall, treatment with statins during pregnancy did not increase the risk of congenital anomalies. There was a small increased risk of cardiac malformations and spontaneous abortions, seen only in cohort studies, mainly with statin exposure in the first trimester. This study suggests that there is a need to re-evaluate the role of statin therapy in pregnant patients in whom there may be a significant benefit, in particular in the second and third trimesters. The risk and benefit of statins treatment during pregnancy need to be evaluated in an individualized approach and every trimester apart.

## Data Availability

The original contributions presented in the study are included in the article/Supplementary Material, further inquiries can be directed to the corresponding author.

## Abbreviations

HMG-CoA, Hydroxymethylglutaryl-coenzyme A; FDA, Federal Drug Administration; RCTs, randomized controlled trials; ACEI, Angiotensin Converting Enzyme inhibitor, ARB, Angiotensin II Receptor Blocker.
