# Supplementary material for: The effect of statins exposure during pregnancy on congenital anomalies and spontaneous abortions: A systematic review and meta-analysis
Source: Front Pharmacol. 2022 Sep 29;13:1003060. doi: 10.3389/fphar.2022.1003060 (PMC9558136; doi:10.3389/fphar.2022.1003060)

## Supplementary material

*Table S1-*
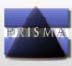
**PRISMA 2020 Checklist**

| **Section and Topic** | **Item #** | **Checklist item** | **Location where item is reported** |
| --- | --- | --- | --- |
| **TITLE** | | |  |
| Title | 1 | Identify the report as a systematic review. | 1 |
| **ABSTRACT** | | |  |
| Abstract | 2 | See the PRISMA 2020 for Abstracts checklist. | 3-4 |
| **INTRODUCTION** | | |  |
| Rationale | 3 | Describe the rationale for the review in the context of existing knowledge. | 5-6 |
| Objectives | 4 | Provide an explicit statement of the objective(s) or question(s) the review addresses. | 6 |
| **METHODS** | | |  |
| Eligibility criteria | 5 | Specify the inclusion and exclusion criteria for the review and how studies were grouped for the syntheses. | 6 |
| Information sources | 6 | Specify all databases, registers, websites, organisations, reference lists and other sources searched or consulted to identify studies. Specify the date when each source was last searched or consulted. | 7 |
| Search strategy | 7 | Present the full search strategies for all databases, registers and websites, including any filters and limits used. | 6-7+table s3 |
| Selection process | 8 | Specify the methods used to decide whether a study met the inclusion criteria of the review, including how many reviewers screened each record and each report retrieved, whether they worked independently, and if applicable, details of automation tools used in the process. | 7-8 |
| Data collection process | 9 | Specify the methods used to collect data from reports, including how many reviewers collected data from each report, whether they worked independently, any processes for obtaining or confirming data from study investigators, and if applicable, details of automation tools used in the process. | 7-8 |
| Data items | 10a | List and define all outcomes for which data were sought. Specify whether all results that were compatible with each outcome domain in each study were sought (e.g. for all measures, time points, analyses), and if not, the methods used to decide which results to collect. | 7-8 |
|  | 10b | List and define all other variables for which data were sought (e.g. participant and intervention characteristics, funding sources). Describe any assumptions made about any missing or unclear information. | 7-8 |
| Study risk of bias assessment | 11 | Specify the methods used to assess risk of bias in the included studies, including details of the tool(s) used, how many reviewers assessed each study and whether they worked independently, and if applicable, details of automation tools used in the process. | 8 |
| Effect measures | 12 | Specify for each outcome the effect measure(s) (e.g. risk ratio, mean difference) used in the synthesis or presentation of results. | 9 |
| Synthesis methods | 13a | Describe the processes used to decide which studies were eligible for each synthesis (e.g. tabulating the study intervention characteristics and comparing against the planned groups for each synthesis (item #5)). | 9 |
|  | 13b | Describe any methods required to prepare the data for presentation or synthesis, such as handling of missing summary statistics, or data conversions. | 8-9 |
|  | 13c | Describe any methods used to tabulate or visually display results of individual studies and syntheses. | 8-9 |
|  | 13d | Describe any methods used to synthesize results and provide a rationale for the choice(s). If meta-analysis was performed, describe the model(s), method(s) to identify the presence and extent of statistical heterogeneity, and software package(s) used. | 8-9 |
|  | 13e | Describe any methods used to explore possible causes of heterogeneity among study results (e.g. subgroup analysis, meta-regression). | 9 |
|  | 13f | Describe any sensitivity analyses conducted to assess robustness of the synthesized results. | 9 |
| Reporting bias assessment | 14 | Describe any methods used to assess risk of bias due to missing results in a synthesis (arising from reporting biases). | 8-9 |
| Certainty assessment | 15 | Describe any methods used to assess certainty (or confidence) in the body of evidence for an outcome. | 9 |
| **RESULTS** | | |  |
| Study selection | 16a | Describe the results of the search and selection process, from the number of records identified in the search to the number of studies included in the review, ideally using a flow diagram. | 10 |
|  | 16b | Cite studies that might appear to meet the inclusion criteria, but which were excluded, and explain why they were excluded. | 10 |
| Study characteristics | 17 | Cite each included study and present its characteristics. | 11 |
| Risk of bias in studies | 18 | Present assessments of risk of bias for each included study. | 11+fig S1+ table S4 |
| Results of individual studies | 19 | For all outcomes, present, for each study: (a) summary statistics for each group (where appropriate) and (b) an effect estimate and its precision (e.g. confidence/credible interval), ideally using structured tables or plots. | 12-13 |
| Results of syntheses | 20a | For each synthesis, briefly summarise the characteristics and risk of bias among contributing studies. | 12-13 |
|  | 20b | Present results of all statistical syntheses conducted. If meta-analysis was done, present for each the summary estimate and its precision (e.g. confidence/credible interval) and measures of statistical heterogeneity. If comparing groups, describe the direction of the effect. | 12-13 |
|  | 20c | Present results of all investigations of possible causes of heterogeneity among study results. | 12-13 |
|  | 20d | Present results of all sensitivity analyses conducted to assess the robustness of the synthesized results. | 11-13 |
| Reporting biases | 21 | Present assessments of risk of bias due to missing results (arising from reporting biases) for each synthesis assessed. | 11+fig S2-S3 |
| Certainty of evidence | 22 | Present assessments of certainty (or confidence) in the body of evidence for each outcome assessed. | ? |
| **DISCUSSION** | | |  |
| Discussion | 23a | Provide a general interpretation of the results in the context of other evidence. | 13-14 |
|  | 23b | Discuss any limitations of the evidence included in the review. | 14-17 |
|  | 23c | Discuss any limitations of the review processes used. | 14-17 |
|  | 23d | Discuss implications of the results for practice, policy, and future research. | 16-17 |
| **OTHER INFORMATION** | | |  |
| Registration and protocol | 24a | Provide registration information for the review, including register name and registration number, or state that the review was not registered. | 1 |
|  | 24b | Indicate where the review protocol can be accessed, or state that a protocol was not prepared. | 1 |
|  | 24c | Describe and explain any amendments to information provided at registration or in the protocol. |  |
| Support | 25 | Describe sources of financial or non-financial support for the review, and the role of the funders or sponsors in the review. | 1 |
| Competing interests | 26 | Declare any competing interests of review authors. | 1 |
| Availability of data, code and other materials | 27 | Report which of the following are publicly available and where they can be found: template data collection forms; data extracted from included studies; data used for all analyses; analytic code; any other materials used in the review. | ? |

*Table S2- MOOSE Checklist for Meta-analyses of Observational Studies*

| **Item No** | **Recommendation** | **Reported on Page No** |
| --- | --- | --- |
| Reporting of background should include | | |
| 1 | Problem definition | 5-6 |
| 2 | Hypothesis statement | 6 |
| 3 | Description of study outcome(s) | 7 |
| 4 | Type of exposure or intervention used | 5-6 |
| 5 | Type of study designs used | 5-6 |
| 6 | Study population | 5-6 |
| Reporting of search strategy should include | | |
| 7 | Qualifications of searchers (eg, librarians and investigators) | 6 |
| 8 | Search strategy, including time period included in the synthesis and key words | 6-7+fig1+table s3 |
| 9 | Effort to include all available studies, including contact with authors | 6-8. 7+fig1+table s3 |
| 10 | Databases and registries searched | 6 |
| 11 | Search software used, name and version, including special features used (eg, explosion) | 6-8 |
| 12 | Use of hand searching (eg, reference lists of obtained articles) | - |
| 13 | List of citations located and those excluded, including justification | 7,10, Table 1, Fig 1 |
| 14 | Method of addressing articles published in languages other than English | - |
| 15 | Method of handling abstracts and unpublished studies | - |
| 16 | Description of any contact with authors | 8 |
| Reporting of methods should include | | |
| 17 | Description of relevance or appropriateness of studies assembled for assessing the hypothesis to be tested | 6-7 |
| 18 | Rationale for the selection and coding of data (eg, sound clinical principles or convenience) | 6-7, Table S3 |
| 19 | Documentation of how data were classified and coded (eg, multiple raters, blinding and interrater reliability) | 6-10 |
| 20 | Assessment of confounding (eg, comparability of cases and controls in studies where appropriate) | 17 |
| 21 | Assessment of study quality, including blinding of quality assessors, stratification or regression on possible predictors of study results | 8,11 |
| 22 | Assessment of heterogeneity | 9 |
| 23 | Description of statistical methods (eg, complete description of fixed or random effects models, justification of whether the chosen models account for predictors of study results, dose-response models, or cumulative meta-analysis) in sufficient detail to be replicated | 9 |
| 24 | Provision of appropriate tables and graphics | Tables 1, Figs 1-6, Figs S1-S10 |
| Reporting of results should include | | |
| 25 | Graphic summarizing individual study estimates and overall estimate | Figs 1-4, Figs S1-S10 |
| 26 | Table giving descriptive information for each study included | Table 1 |
| 27 | Results of sensitivity testing (eg, subgroup analysis) | Table S4+figS1  12-13 |
| 28 | Indication of statistical uncertainty of findings | - |

*Table S3-Search sequences that were used in the different databases.*

| Search sequence | Database |
| --- | --- |
| (((("pregnan*"[Title/Abstract] OR "reproduction"[Title/Abstract] OR "conception"[Title/Abstract] OR "prenatal development"[Title/Abstract] OR "Embryology"[Title/Abstract] OR "prenatal exposure"[Title/Abstract] OR "prenatal drug exposure"[Title/Abstract] OR "intrauterine drug exposure"[Title/Abstract] OR "maternal drug exposure"[Title/Abstract] OR "foetal drug exposure"[Title/Abstract] OR "fetus exposure"[Title/Abstract] OR "prenatal environment"[Title/Abstract] OR "reproductive interference"[Title/Abstract] OR "child bearing"[Title/Abstract] OR "childbearing" [Title/Abstract] OR "childbirth"[Title/Abstract] OR "gestation*"[Title/Abstract] OR "Gravidity"[Title/Abstract] OR "labor"[Title/Abstract] OR "labour"[Title/Abstract] OR "Parturient"[Title/Abstract] OR "trimester"[Title/Abstract] OR "midtrimester"[Title/Abstract] OR "maternal"[Title/Abstract] OR "fetus"[Title/Abstract] OR "prenatal disorder"[Title/Abstract] OR ("Pregnancy"[MeSH Terms] OR "Embryology"[MeSH Terms] OR "Pregnant Women"[MeSH Terms] OR "Gravidity"[MeSH Terms] OR "Gestational Age"[MeSH Terms] OR "Parity"[MeSH Terms] OR "Pregnancy Trimesters"[MeSH Terms])) OR "pregnan*"[Title/Abstract] OR "[Title/Abstract] OR "prenatal development"[Title/Abstract] OR "embryology"[Title/Abstract] OR "prenatal exposure"[Title/Abstract] OR "prenatal drug exposure"[Title/Abstract] OR "intrauterine drug exposure"[Title/Abstract] OR "maternal drug exposure"[Title/Abstract] OR "foetal drug exposure"[Title/Abstract] OR "fetus exposure"[Title/Abstract] OR "prenatal environment"[Title/Abstract] OR "reproductive interference"[Title/Abstract] OR "child bearing"[Title/Abstract] OR "childbearing"[Title/Abstract] OR "childbirth"[Title/Abstract] OR "gestation*"[Title/Abstract] OR "gravidity"[Title/Abstract] OR "labor"[Title/Abstract] OR "labour"[Title/Abstract] OR "Parturient"[Title/Abstract] OR "trimester"[Title/Abstract] OR "midtrimester"[Title/Abstract] OR "maternal"[Title/Abstract] OR "fetus"[Title/Abstract] OR "prenatal disorder"[Title/Abstract]AND ("Hydroxymethylglutaryl coenzyme A reductase inhibitor"[Title/Abstract] OR "hydroxymethylglutaryl coa reductase inhibitors"[Title/Abstract] OR "hydroxymethylglutaryl coa reductase inhibitors"[Title/Abstract] OR "hmg coa reductase inhibitors"[Title/Abstract] OR "HMG CoA reductase inhibitor"[Title/Abstract] OR "hmg coa reductase inhibitors"[Title/Abstract] OR "hmg coenzyme a reductase inhibitor"[Title/Abstract] OR "anticholesteremic agent"[Title/Abstract] OR "hypocholesterolemic agent"[Title/Abstract] OR "statin*"[Title/Abstract] OR "vastatin"[Title/Abstract] OR "simvastatin"[Title/Abstract] OR "pitavastatin"[Title/Abstract] OR "lovastatin"[Title/Abstract] OR "Fluvastatin" [Title/Abstract] OR "pravastatin"[Title/Abstract] OR "atorvastatin"[Title/Abstract] OR "rosuvastatin"[Title/Abstract] OR "cerivastatin"[Title/Abstract] OR "compactin" [Title/Abstract] OR "crilvastatin"[Title/Abstract] OR "dalvastatin"[Title/Abstract] OR "Mevinolin"[Title/Abstract] OR "mevinolinic acid"[Title/Abstract] OR "monacolin" [Title/Abstract] OR ("hydroxymethylglutaryl coa reductase inhibitors"[MeSH Terms] OR "hydroxymethylglutaryl coa reductase inhibitors"[Pharmacological Action]))) NOT ("animals"[MeSH Terms] NOT "humans"[MeSH Terms])) NOT ("Systematic Review"[Publication Type] OR "meta-analysis"[Publication Type] OR "Review"[Publication Type] OR "Editorial"[Publication Type] OR "Comment"[Publication Type])) NOT "animals"[MeSH Terms] NOT "humans"[MeSH Terms] | PubMed |
| ((((pregnan*:ti,ab,kw OR reproduction:ti,ab,kw OR conception:ti,ab,kw OR 'prenatal development':ti,ab,kw OR embryology:ti,ab,kw OR 'prenatal exposure':ti,ab,kw OR 'prenatal drug exposure':ti,ab,kw OR 'intrauterine drug exposure':ti,ab,kw OR 'maternal drug exposure':ti,ab,kw OR 'foetal drug exposure':ti,ab,kw OR 'fetus exposure':ti,ab,kw OR 'prenatal environment':ti,ab,kw OR 'reproductive interference':ti,ab,kw OR 'child bearing':ti,ab,kw OR childbearing:ti,ab,kw OR childbirth:ti,ab,kw OR gestation*:ti,ab,kw OR gravidity:ti,ab,kw OR labor:ti,ab,kw OR labour:ti,ab,kw OR parturient:ti,ab,kw OR trimester:ti,ab,kw OR midtrimester:ti,ab,kw OR maternal:ti,ab,kw OR fetus:ti,ab,kw OR 'prenatal disorder':ti,ab,kw) OR ('pregnancy'/exp OR 'prenatal development'/exp OR 'prenatal disorder'/exp OR 'pregnant woman'/exp)) AND (('hydroxymethylglutaryl coenzyme a reductase inhibitor':ti,ab,kw OR 'hydroxymethylglutaryl coa reductase inhibitors':ti,ab,kw OR 'hydroxymethylglutaryl-coa reductase inhibitors':ti,ab,kw OR 'hmg-coa-reductase inhibitors':ti,ab,kw OR 'hmg coa reductase inhibitor':ti,ab,kw OR 'hmg coa reductase inhibitors':ti,ab,kw OR 'hmg coenzyme a reductase inhibitor':ti,ab,kw OR 'anticholesteremic agent':ti,ab,kw OR 'hypocholesterolemic agent':ti,ab,kw OR statin*:ti,ab,kw OR vastatin:ti,ab,kw OR simvastatin:ti,ab,kw OR pitavastatin:ti,ab,kw OR lovastatin:ti,ab,kw OR fluvastatin:ti,ab,kw OR pravastatin:ti,ab,kw OR atorvastatin:ti,ab,kw OR rosuvastatin:ti,ab,kw OR bervastatin:ti,ab,kw OR cerivastatin:ti,ab,kw OR compactin:ti,ab,kw OR crilvastatin:ti,ab,kw OR dalvastatin:ti,ab,kw OR mevinolin:ti,ab,kw OR 'mevinolinic acid':ti,ab,kw OR monacolin:ti,ab,kw) OR 'hydroxymethylglutaryl coenzyme a reductase inhibitor'/exp)) NOT ('animals'/exp NOT 'humans'/exp)) NOT ('review'/exp OR 'editorial'/exp OR 'note'/exp OR 'conference review'/exp OR 'systematic review'/exp OR 'meta analysis'/exp) AND [2020-2021]/py | Embase |
| obstetric OR obstetrical OR pregnant OR pregnancy OR reproduction OR conception OR "prenatal development" OR embryology OR "prenatal exposure" OR "prenatal drug exposure" OR "intrauterine drug exposure" OR "maternal drug exposure"OR "foetal drug exposure" OR "fetus exposure" OR "prenatal environment" OR "reproductive interference" OR "child bearing" OR childbearing OR childbirth OR gestation OR gravidity \| "Hydroxymethylglutaryl coenzyme A reductase inhibitor" OR "hydroxymethylglutaryl coa reductase inhibitors" OR "Hydroxymethylglutaryl-CoA reductase inhibitors" OR "HMG-CoA-reductase inhibitors" OR "HMG CoA reductase inhibitor" OR "HMG CoA reductase inhibitors" OR "hmg coenzyme a reductase inhibitor" OR "anticholesteremic agent" OR "hypocholesterolemic agent" OR statin OR statins OR vastatin OR simvastatin OR pitavastatin OR lovastatin OR Fluvastatin OR pravastatin OR atorvastatin OR rosuvastatin OR bervastatin OR cerivastatin OR compactin OR crilvastatin OR dalvastatin OR Mevinolin OR "mevinolinic acid" OR monacolin. | Clinical trials |
| pregnan* OR reproduction OR conception OR "prenatal development" OR embryology OR "prenatal exposure" OR "prenatal drug exposure" OR "intrauterine drug exposure" OR "maternal drug exposure" OR "foetal drug exposure" OR "fetus exposure" OR "prenatal environment" OR "reproductive interference" OR "child bearing" OR childbearing OR childbirth OR gestation* OR gravidity OR labor OR labour OR Parturient OR trimester OR midtrimester OR maternal OR fetus OR "prenatal disorder"):ti,ab,kw AND ("Hydroxymethylglutaryl coenzyme A reductase inhibitor" OR "hydroxymethylglutaryl coa reductase inhibitors" OR "Hydroxymethylglutaryl-CoA reductase inhibitors" OR "HMG-CoA-reductase inhibitors" OR "HMG CoA reductase inhibitor" OR "HMG CoA reductase inhibitors" OR "hmg coenzyme a reductase inhibitor" OR "anticholesteremic agent" OR "hypocholesterolemic agent" OR statin* OR vastatin OR simvastatin OR pitavastatin OR lovastatin OR Fluvastatin OR pravastatin OR atorvastatin OR rosuvastatin OR bervastatin OR cerivastatin OR compactin OR crilvastatin OR dalvastatin OR Mevinolin OR "mevinolinic acid" OR monacolin):ti,ab,kw (Word variations have been searched)) | Cochrane |

| *Table S4: List of malformations–cases (where available)* | | |
| --- | --- | --- |
| ***Malformation*** | ***Exposure*** | ***Study, year*** |
| Unspecified anomaly of the heart  Ventricular septal defect,  Unspecified defect of septal closure  Other specified anomaly of heart,  Ostium secundum type atrial septal  defect | Lovastatin  Simvastatin  Atorvastatin | Ofori, 2007 |
| Cervical soft tissue mass (minor anomaly which was surgically removed) | Atorvastatin, simvastatin,  pravastatin, rosuvastatin | Taguchi, 2008 |
| Missing middle phalanx, right ring finger, Dilated left renal pelvis, Cutaneous angioma.  Sacrococcygeal teratoma, hip joint deformity Urethral obstruction, fetal death.  Trisomy 21, Fetal cardiomegaly (sever),   fetal arrhythmia (fetal death at 27 weeks of gestation)  Haemangioma on neck and left temple.  Inguinal hernia, Choroid plexus cyst  Hip luxation, Inguinal hernia, Sacral pit, Choroid plexus cyst, Umbilical hernia (surgery at age of 3 years), Congenital hydrocele | Major birth defects  Atorvastatin  Pravastatin  Rosuvastatin  Minor birth defects and development disorders  Pravastatin  Simvastatin | Winterfeld, 2013 |
| Central nervous system malformations, Cardiac malformations, Respiratory malformations, Cleft palate and lip, Gastrointestinal malformations, Genitourinary malformations, Musculoskeletal malformations, Other * There are no details regarding the specific malformations | Simvastatin, lovastatin, pravastatin, fluvastatin,  atorvastatin, cerivastatin, and rosuvastatin | Bateman, 2015 |
| Hypospadias, Coarctation of aorta | Pravastatin | Constantine, 2016 |
| Major malformations *There are no details regarding the specific malformations | Simvastatin, atorvastatin  cerivastatin, rosuvastatin, pravastatin, fluvastatin and combination | McGrogan, 2017 |
| Cardiac anomalies including atrial septal defect (ASD), ventricular septal defect (VSD), Conotruncal defect (includes tetralogy of Fallot, transposition of the aorta, and truncus arteriosus), Single ventricle physiology (includes hypoplastic left ventricle and common ventricle), patent ductus arteriosus (PDA), Coarctation of the aorta | Atorvastatin, lovastatin, pravastatin, simvastatin | Ming-Sum Lee, 2018 |
| No congenital anomalies were detected | Pravastatin | Lefkou, 2016 |
| No congenital anomalies were detected | Pravastatin | Deviana, 2019 |
| Small intracerebral cyst, intermittent cardiac murmur, hypospadias | Pravastatin | Ahmed A, 2019 |
| There are no details regarding the specific malformations | Atorvastatin, Rosuvastatin, Lovastatin, Simvastatin, Fluvastatin, Pravastatin, combination | Chung Chang, 2021 |
| No congenital anomalies were detected in pravastatin group | Pravastatin | Costantine, 2021 |

*In yellow are marked cardiac malformations

*Table S5- key outcomes of the included trials*

|  | **Congenital anomalies** | | | | | **Cardiac malformation** | | | | | **Spontaneous abortions** | | | | |
| --- | --- | --- | --- | --- | --- | --- | --- | --- | --- | --- | --- | --- | --- | --- | --- |
|  | Treated events/ total treated | Untreated events/ total untreated | OR (CI 0.95) | Lower limit | Upper limit | Treated events/ total treated | Untreated events/ total untreated | OR (CI 0.95) | Lower limit | Upper limit | Treated events/ total treated | Untreated events/ total untreated | OR (CI 0.95) | Lower limit | Upper limit |
| **Ofori, 2007** |  |  | 0.36 | 0.06 | 2.18 | 3/64 | 3/67 |  |  |  | 52/153 | 29/106 |  |  |  |
| **Taguchi, 2008** | 1/46 | 1/52 |  |  |  |  |  |  |  |  | 14/64 | 11/64 |  |  |  |
| **Winterfeld,2013** | 8/197 | 6/224 |  |  |  | 1/197 | 1/224 |  |  |  |  |  | 1.36 | 0.63 | 2.93 |
| **Bateman, 2015** |  |  | 1.07 | 0.85 | 1.37 |  |  | 1.25 | 0.93 | 1.7 |  |  |  |  |  |
| **McGrogan,2017** | 5/156 | 48/1701 |  |  |  |  |  |  |  |  |  |  | 1.64 | 1.1 | 2.46 |
| **Costantine,2016** | 2/10 | 2/10 |  |  |  | 1/10 | 0/10 |  |  |  |  |  |  |  |  |
| **Lefkou, 2016** | 0/11 | 0/11 |  |  |  | 1/10 | 0/10 |  |  |  |  |  |  |  |  |
| **Ming-Sum,2018** |  |  | 2.5 | 1.3 | 4.9 |  |  | 2.5 | 1.3 | 4.9 |  |  |  |  |  |
| **Ahmed, 2019** | 3/30 | 2/32 |  |  |  | 1/30 | 0/32 |  |  |  |  |  |  |  |  |
| **Soraya, 2019** | 0/18 | 0/15 |  |  |  | 0/18 | 0/15 |  |  |  |  |  |  |  |  |
| **Costantine,2021** | 0/10 | 2/10 |  |  |  | 0/10 | 1/10 |  |  |  |  |  |  |  |  |
| **ChungChang,2021** |  |  | 1.24 | 0.81 | 1.9 |  |  |  |  |  |  |  |  |  |  |

*Table S6: Risk of bias summary: review authors' judgments about each risk of bias item for each included cohort study*

| **Newcastle-Ottawa quality assessment scale** | | | | |
| --- | --- | --- | --- | --- |
| NOS score | Outcome | Comparability | Selection | Author, year, Study |
| 8 | ** | ** | **** | ***Ofori, 2007*** |
| 8 | *** | ** | *** | ***Taguchi, 2008*** |
| 9 | *** | ** | **** | ***Winterfeld, 2013*** |
| 9 | *** | ** | **** | ***Bateman, 2015*** |
| 7 | *** | * | *** | ***Lefkou E, 2016*** |
| 6 | *** | * | ** | ***McGrogan, 2017*** |
| 8 | *** | ** | *** | ***Ming-Sum Lee, 2018*** |
| 9 | *** | ** | **** | ***Mendoza M, 2020*** |
| 9 | *** | *** | *** | ***Chung Chang, 2021*** |

*Figure S1-Risk of bias summary: review authors' judgments about each risk of bias item for each included RCT study.*


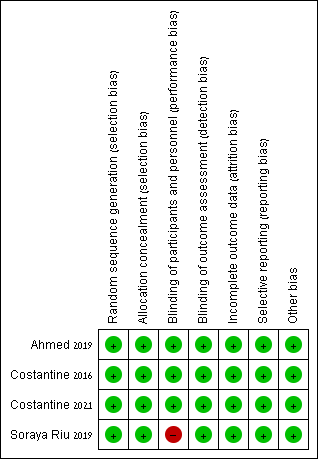


*Figure S2: Funnel plot for publication bias assessment (congenital malformation)*

**

*Figure S3: Funnel plot for publication bias assessment (cardiac anomalies)*

**

*Figure S4*

*The Odd ratios for cardiac anomalies following statins treatment versus control treatment- sensitivity analysis:* ******

***Meta-regression***

*Figure S5- Meta-regression analysis results evaluating the correlation between lipophilic statins (%) and congenital malformations.*

**

*Figure S6- Meta-regression analysis results evaluating the correlation between lipophilic statins (%) and cardiac malformations*

***Figure S7- Meta-regression analysis results evaluating the difference in diabetes prevalence between treatment group and the association to congenital malformations.*

*Figure S8- Meta-regression analysis results evaluating the difference in diabetes prevalence between treatment group and the association to cardiac malformations*

*Figure S9- Meta-regression analysis results evaluating the* difference in hypertension *prevalence between treatment group and the association to congenital malformations*

*S10- Meta-regression analysis results evaluating the* difference in *prevalence between treatment group and the association to cardiac malformations*

*Table S7: Percentage of cases where ACEI's, ARB's and anti-epileptic drugs*

*used among studies that were included in cardiac malformation analysis*

| **ACEI\ARBS use** | | | **Antiepileptic drugs use** | | | | **Study, year** | | |
| --- | --- | --- | --- | --- | --- | --- | --- | --- | --- |
| % case | % control | | % case | % control | | |  | | |
| 2.13%  )Antihypertensive drugs - which include ACEI \ ARBS but there is no specific percentage for these families( | 4.57% (Antihypertensive drugs - which include ACEI \ ARBS but there is no specific percentage for these families) | | Women who used category X drugs (including carbamazepine, phenytoin, valproic acid) were excluded. | | | | **Ofori, 2007** | | |
| Women with any exposures to known human teratogens were excluded. | | | | | | | **Taguchi, 2008** | | |
| For all malformations: 2.03% (4/197)  For major malformations 1.52%: (3/197) | 0% | | 0% | 0% | | | **Winterfeld, 2013** | | |
| Also included in propensity score mode exposure to potentially teratogenic drugs during first trimester (**including angiotensin**  **converting enzyme inhibitors** | | | Excluded pregnancies that were exposed to teratogenic drugs known as antineoplastic agents. | | | | **Bateman, 2015** | | |
| - | - | | Women with epilepsy were excluded | | | | **Costantine, 2016** | | |
| - | - | | - | - | | | **Lefkou, 2016** | | |
| ACEI: 25.98%  ARBS: 6.05% | ACEI: 5.11%  ARBS: 1.63% | | 4.27%  Epilepsy medication | 1.66%  Epilepsy medication | | | **McGrogan, 2017** | | |
| - | - | | - | - | | | **Lee, 2018** | | |
| - | - | | - | - | | | **Ahmed, 2019** | | |
| - | - | | - | - | | | **Soraya, 2019** | | |
| Women that used teratogenic drugs during pregnancy were excluded | | | Women with epilepsy were excluded | | | | **Chung Chang, 2021** | | |
| participation with another intervention that could influence the outcomes were excluded | | | | | | | **Costantine, 2021** | | |

*Figure S11- The Odd ratios for congenital anomalies following statins treatment versus control treatment sensitivity analysis without Batemen study*


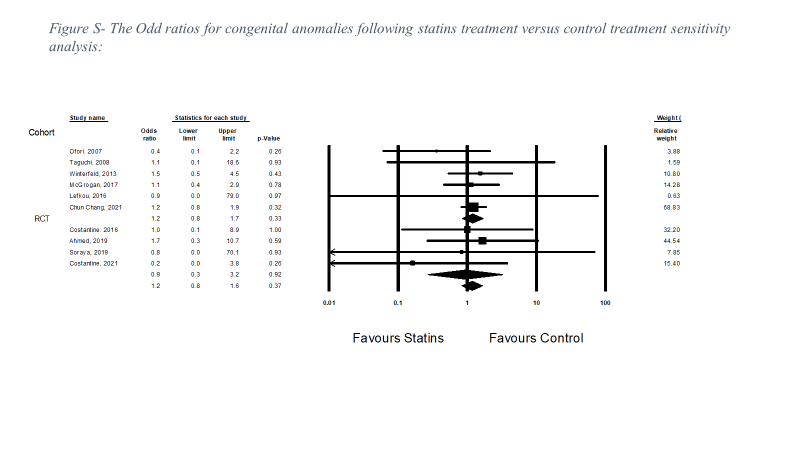

Supplement: Supplementary file 1 [file DataSheet1.docx]
